# Pain Medicine and the Opioid Crisis: Time to Own Our Responsibility!

**DOI:** 10.1002/ejp.70263

**Published:** 2026-04-04

**Authors:** Duncan Ritchie, Patrice Forget

**Affiliations:** ^1^ Department of Anaesthesia, NHS Grampian Aberdeen UK; ^2^ Institute of Education in Healthcare and Medical Sciences University of Aberdeen, Foresterhill Aberdeen UK; ^3^ Aberdeen Centre for Musculoskeletal Health (Epidemiology Group) Institute of Applied Health Sciences, University of Aberdeen, Health Sciences Building Aberdeen UK; ^4^ Pain AND Opioids After Surgery (PANDOS) European Society of Anaesthesiology and Intensive Care (ESAIC) Research Group (ID ESAIC_RG_PAND) Brussels Belgium; ^5^ Imagine UR UM 103 Montpellier University, Anesthesia Critical Care, Emergency and Pain Medicine Division, Nîmes University Hospital Nîmes France

**Keywords:** opioid use disorders, opioids, pain management

## Abstract

Pain remains a major unmet health need. Rising opioid prescribing has not reduced population pain and has directly contributed to increased morbidity and mortality. Opioid use disorder (OUD) and pain frequently coexist. Many people with OUD experience acute and chronic pain and a substantial proportion of patients prescribed opioids develop OUD. We have contributed to this problem. In this Position Paper, we defend the idea that we must urgently redefine pain medicine as a frontline discipline in opioid use disorder prevention and recovery. Pain management must be reframed as part of the solution for people with or at risk of OUD. Core principles include: early identification of OUD and psychiatric comorbidity, routine incorporation of trauma‐informed, stigma‐reducing communication and shared decision‐making; continuation of opioid agonist therapy during acute care; use of tailored multimodal, opioid‐sparing analgesia; and perioperative planning with addiction and primary care follow‐up. At a system level, we recommend multidisciplinary opioid stewardship, clear limits on opioid indication, dose and duration, diversion‐mitigation strategies and policy changes such as reduced pack sizes and limiting indications for prolonged‐released opioids. Research priorities include qualitative studies of lived experience, inclusion of patient‐reported outcomes in trials and big‐data analyses of prescribing to identify targets for intervention. Integrating clinical best practice, stigma‐aware care and system reforms can reduce harm while ensuring equitable, effective pain relief for people with pain and those with or at risk of OUD.

**Significance:**

This position paper synthesises clinical, psychosocial and system‐level strategies to manage pain in people with or at risk of OUD. It emphasises our unique responsibility by learning from failures, and constructively sees the future. Treatment continuity, trauma‐informed and stigma‐reducing communication, integrated care, interdisciplinary coordination and opioid stewardship policies are no longer optional. These recommendations aim to reduce harms, improve pain and mental‐health outcomes and guide clinicians and policymakers toward better and more equitable care.

## 1 Introduction

Pain remains a significant problem. Inadequate access, limited effectiveness, imperfect safety and important societal costs continue to justify significant resource allocation, aiming at improving pain management (Azevedo et al. 2016; Darnall et al. 2023; Forget and Hauser 2023; Institute of Medicine (US) Committee on Advancing Pain Research, Care, and Education 2011). The massive increase in opioid prescriptions, during the last twenty years, in many countries, has not solved the problem. And even more, we may have contributed to the diversion of opioids in some countries, and to the stigmatisation of people using opioids, in most. In this Position Paper, we defend the idea that we must urgently redefine pain medicine as a frontline discipline in opioid use disorder prevention and recovery, where pain management is a solution rather than a problem, in particular in people with substance use disorders.

Substance use disorders, and in particular opioid use disorder (OUD), remain globally problematic. Recent data from the European Union Drug Agency show an increase in the incidence of drug‐induced deaths, with an opioid involved in the vast majority of cases (European Union Drugs Agency 2025). While most of these are attributable to the use of illicit opioids, such as heroin, data from North America clearly shows that OUD is also prevalent in people taking prescribed opioids (Kolodny and Bohler 2024). The OPERA study in Canada has analysed the risk of initiation of drug injection in those prescribed an opioid. Key risk factors include young age and the chronic prescription of high dose opioids (Wilton et al. 2021). Importantly, however, when looking at the entire cohort, the majority of patients were in the (much larger) acute opioid prescription group, rather than in the episodic or chronic prescription groups (Forget 2021). While the acute prescription may not be the cause, it is clear that it often precedes the development of problematic opioid use.

Taking that into account, it is morally indefensible not to integrate SUD prevention (primary and secondary) into all pain management education, practice and policy. This approach is essential for clinicians offering pain management.

Pain is experienced by the vast majority of opioid users. This includes illicit opioid users, who notably suffer from a high incidence of musculoskeletal pain (Kim et al. 2025). Pain is not a protective factor against the risk of developing SUD, and offering opioid agonist treatment (OAT), such as methadone or buprenorphine, does not necessarily lead to the desire to reduce or stop illicit opioid use. Even if engaged in OAT, 60% continue to suffer from pain, frequently associated with poorer mental health (Yang et al. 2024). In short, if pain accompanies the journey of people with OUD, pain specialists must be trained to prevent and recognise SUD as part of their core competency.

If pain accompanies the journey of people with OUD, could OUD also accompany the journey of people with chronic pain? While the above‐mentioned figures are similar in Europe and North America, major differences reside in the heterogeneous prescription rates and issues related to opioids, including a generally much lower, although highly variable, incidence of opioid‐related deaths. A large European survey showed that opioid prescription rates significantly increased across Europe, with most countries more than doubling their prescription rates during the decade preceding 2015 (Häuser et al. 2021). A recent meta‐analysis, also including a significant number of European studies, suggested that prevalence of aberrant behaviour regarding opioid use may be in the order of 22% (95% confidence interval—95% CI: 17%–27%). Signs and symptoms of dependence and OUD are displayed in 30% (95% CI: 22%–38%), with 9% (95% CI: 6%–15%) meeting the criteria for dependence and OUD, according to established diagnostic codes (Thomas et al. 2024). It is important to note, at this stage, that these criteria not only cover different clinical realities from a patient perspective (dependence criteria are mostly based on physical symptoms, even if opioids may be taken to relieve them, while OUD relates to a problematic pattern of substance use leading to impairment or distress), but are also expected when an opioid prescription becomes chronic (in particular, physical dependence). The incapacity to meet social or professional role may be related to side effects, but also to the pain condition. This complicates the picture and highlights the ethical obligation to assess SUD risk and prevention strategies with every opioid prescription, including a discussion when, while introducing opioids, problems may outweigh benefits.

## 2 Approaching Pain Management in OUD

Patients with OUD presenting with acute or chronic pain represent a population with unique challenges. Chronic opioid use results in widespread neuroadaptations that contribute to tolerance, dependence and altered pain processing. At the receptor level, μ‐opioid receptors undergo desensitisation and internalisation, diminishing their effectiveness. Different opioids vary in their propensity to induce internalisation, which may influence their potential for dependence. Agents like methadone and buprenorphine, used for OAT, tend to induce greater internalisation compared to morphine, potentially offering protective effects (Mehta and Langford 2009).

Cellular adaptations predominantly involve the cAMP signalling cascade. Opioids acutely suppress adenyl cyclase activity and reduce cAMP levels. With chronic use, compensatory upregulation restores cAMP, leading to altered neurotransmitter release. Upon opioid withdrawal, the sudden loss of inhibition on adenyl cyclase can precipitate withdrawal symptoms. Systemic neural network adaptations also emerge, including changes in opioid‐sensitive circuits and glial‐neuronal interactions. For instance, despite μ‐opioid agonists not acting directly on dopaminergic neurons, adaptations in this pathway have been implicated in addiction (Mehta and Langford 2009).

Patients with OUD, particularly those who use illicitly obtained opioids, are often subjects of stigma, with healthcare staff frequently harbouring implicit biases against these patients. These biases can influence clinical decision‐making, often resulting in the under‐treatment of pain (Van Boekel et al. 2013). This is not acceptable, and can create a damaging feedback loop: patients, aware of potential judgement or accusations of drug‐seeking, may under‐report pain or avoid seeking care altogether. This reluctance can exacerbate both physical suffering and psychological distress, leading to worsening of their underlying condition and potentially driving continued or escalated drug use. Pain medicine must lead stigma reduction as a standard, not an option, including reflective practice on bias and communication strategies that reaffirm patient dignity and promote shared decision‐making.

OUD seldom exists in isolation. Psychiatric comorbidities such as depression, anxiety and post‐traumatic stress disorder (PTSD) are highly prevalent among individuals with OUD (Jones and McCance‐Katz 2019; Volkow et al. 2016). These conditions not only heighten the subjective experience of pain (Goesling et al. 2013) but also complicate the management of both OUD and pain. For instance, PTSD has been associated with hyperalgesia and altered pain thresholds (Defrin et al. 2008), while depression may diminish treatment engagement or exacerbate somatic symptoms (Bair et al. 2003). Chronic pain itself can lead to the development of depression through various shared molecular mechanisms (Meda et al. 2022).

A comprehensive approach, in this context, must include mental health and OUD screening, integrated care pathways involving substance use psychiatry, and non‐opioid, non‐pharmacological pain therapies, including cognitive behavioural therapy (CBT) and mindfulness‐based interventions, both of which have shown efficacy in improving pain and reducing substance use in this population (Cherkin et al. 2016; Garland et al. 2020).

## 3 Acute Pain Management

In the elective setting, preoperative assessment provides an opportunity to identify patients with OUD and develop a plan for perioperative pain management in conjunction with the patient. Targets at this stage could include establishing rapport and trust, current opioid use including OAT and discussions around realistic expectations for pain management. Pre‐emptive involvement with primary care and substance use services to plan for post‐discharge care can be initiated.

In patients utilising opioid agonist therapy, these should be continued perioperatively (Vadeghani et al. 2024). Previous recommendations suggested interruption to partial agonist therapy, namely buprenorphine, to minimise competition with full agonists being used for analgesia, such as morphine. Vadeghani et al. (2024) found no increased risk of harm in continuing buprenorphine while interrupting therapy is associated with increased risk of relapse, opioid withdrawal and amplification of chronic pain.

Using a multimodal approach to analgesia in patients with OUD is particularly important. This of course extends to all patients, where an opioid sparing approach may avoid the acute opioid exposure placing patients at risk of later developing OUD. Standard agents commonly used for all patients, such as Paracetamol and NSAIDs, should be used where not contraindicated. Locoregional anaesthetic techniques should be utilised where appropriate, with consideration given to placement of catheters for continued analgesia beyond the immediate perioperative period. Low‐dose ketamine (0.15–0.5 mg/kg bolus injection and/or 0.002–0.25 mg/kg/h infusion) has been shown to reduce postoperative pain, opioid requirements and opioid related adverse effects (Meyer‐Frießem et al. 2022). The use of α2‐agonists, Magnesium and lidocaine are all suggested as beneficial in multiple reviews.

Clonidine, an α2‐agonist, has also demonstrated utility in managing the autonomic symptoms of opioid withdrawal. By reducing sympathetic outflow, clonidine alleviates symptoms such as tachycardia, hypertension, sweating and agitation. While it does not address opioid cravings or the full spectrum of withdrawal distress, it can be an effective non‐opioid adjunct in patients withdrawing from opioids.

## 4 Prevention of OUD

As introduced above, it is known that acute opioid prescriptions often precede the development of problematic opioid use and OUD. In addition to this, opioid‐related deaths remain particularly problematic in people with acute conditions or recently discharged from acute care. Using the data from the national naloxone Scottish programme, we have recently identified a cluster of opioid‐related deaths, with one in four happening during the first three months after hospital discharge (Adams et al. 2025). The management of pain and OUD, separately and together, must adopt an integrated, system‐wide, pain OUD prevention strategy, in addition to the patient level management discussed above. As indicated above, this may start in the acute phase with using multimodal, opioid sparing analgesia in opioid naive patients to reduce the risk of OUD further down the road.

A recent international Delphi we initiated (Forget et al. 2022), describes the priorities for system level policy development in relation to opioid use. Key recommendations include the use of a multidisciplinary opioid stewardship steering committee in relation to acute pain management. In a hospital setting, this role can be fulfilled by an acute pain management service. There should be policies surrounding the safe use of drugs at risk of the development of dependence. These include strategies to address the risks of drug diversion. We suggest providing guidelines on maximum doses and duration of treatment for high‐risk medications such as opioids. A notable development in this area is the recent removal of postoperative pain management as an indication for prolonged release opioids (Medicines and Healthcare products Regulatory Agency 2025). One system level change in the future should also include the reduction in pack size of opioids (Adams et al. 2024). In patients with chronic pain, the initiation of opioids should be avoided. This is supported by groups such as the International Association for the Study of Pain (Soliman et al. 2025).

## 5 Looking to the Future

Pain medicine must evolve into an SUD‐aware practice, with prevention as a central goal both for the clinician and the researcher. The future of treatment should focus both on careful, thoughtful prescribing of multimodal analgesia as described above and on expanding interprofessional collaboration (Vadeghani et al. 2024). This collaboration should include the patient at its heart and not only be shaped by the tenets of shared decision making but also those of education. This naturally includes between members of the healthcare team and patient education. We should not forget that patient education provides an opportunity for a two‐way street. The clinician can learn a great deal from the patient as the teacher. This also holds true for the researcher. Future avenues could explore both qualitative studies to explore the lived experience of patients with OUD and the inclusion of patient reported outcome measures including quality of life in future trials. Observational studies also have a role, our group intends to explore “big data” such as whole health system prescribing. This is promising both in describing current practices and in seeking new targets for research and development, which may not be readily visible.

## 6 Conclusion

Opioid use disorder and pain are intricately linked. If opioid use and lack of access may have complicated pain management, we as prescribers have also caused OUD. Ignoring the dependence dimension of pain care is no longer acceptable. Understanding this complex relationship is essential for delivering equitable, compassionate and effective care. Whether the origin of opioid exposure is through illicit use or a well‐intentioned prescription for acute pain, clinicians must be equipped to recognise the continuum of risk. Patients with OUD are patients with pain, as pain medicine clinicians we must own our responsibility to manage these overlapping problems. OUD does not arise in isolation; it frequently coexists with pain, psychiatric illness and socio‐economic adversity.

Effective management must integrate multimodal analgesia, continuity of opioid agonist therapy during hospitalisation, stigma‐aware communication and collaborative pathways. Preventative strategies must extend beyond individual prescribers and encompass systemic reforms, such as opioid stewardship committees, revised prescribing guidelines and packaging regulations.

## Author Contributions

All the authors conceptualised the work, wrote together the manuscript, reviewed, edited and approved the final version and have read and confirmed meeting the ICMJE criteria for authorship.

## Funding

The authors have nothing to report.

## Conflicts of Interest

D.R. declares no conflicts of interest. P.F. is supported by the European Society of Anaesthesiology and Intensive Care (ESAIC) for the Pain and Opioids after Surgery (PANDOS) and the Euro‐Periscope Research Groups (IDs ESAIC_GR_2021_PF, ESAIC_RG_PAND and ESAIC_RG_EP), and received advisory board/speaker fees from Grunenthal and GE Healthcare.
